# Obstructive hydrocephalus of uncommon etiology: case report and neurosurgical management of aqueductal web presenting in adolescence

**DOI:** 10.1007/s00381-024-06645-9

**Published:** 2024-11-01

**Authors:** Aseel Masarwy, Christopher Watterson, Andre Boyke, David Bonda, Moise Danielpour

**Affiliations:** 1 Neurosurgery Department, Cedars Sinai Medical Center, Los Angeles, CA USA; 2 Radiology Department, Cedars Sinai Medical Center, Los Angeles, CA USA

**Keywords:** Hydrocephalus, True-FISP MRI, Endoscopic third ventriculostomy

## Abstract

**Introduction:**

Aqueductal webs are a rare cause of obstructive hydrocephalus. Accurate diagnosis and intervention can prevent neurological complications.

**Case presentation:**

Herein, we describe a case of a child presenting with headaches and vomiting. Magnetic resonance imaging (MRI) revealed obstructive tri-ventricular hydrocephalus caused by an aqueductal web. Endoscopic third ventriculostomy (ETV) was successfully performed to restore cerebrospinal fluid (CSF) flow.

**Conclusion:**

This case underscores the importance of phase-contrast and T2-weighted cinematic magnetic resonance imaging of cerebrospinal fluid flow for diagnosis of aqueductal webs. These modalities provide valuable insights into CSF dynamics and guidance of appropriate neurosurgical intervention.

## Introduction

Aqueductal webs are a rare cause of obstructive hydrocephalus resulting in elevated intracranial pressure. Phase-contrast and T2-weighted cinematic magnetic resonance imaging of cerebrospinal fluid flow can improve diagnosis and guide appropriate management. We present a case of a child presenting with obstructive tri-ventricular hydrocephalus caused by an aqueductal web.

## Case presentation

A 14-year-old male with no significant past medical history presented to our institution with severe early morning headache. He reported several months of intermittent nocturnal and early morning headaches and vomiting. These initially occurred once a month and lasted several hours, but eventually increased to daily, unremitting headaches. A CT scan demonstrated obstructive supratentorial hydrocephalus. There was no mass, hemorrhage, or midline shift on the CT scan.

The patient underwent initial placement of external ventricular drain (EVD) for cerebrospinal fluid (CSF) diversion, without complication, followed by magnetic resonance imaging (MRI) utilizing cardiac-gated true fast imaging with steady-state precession (TrueFISP) imaging.

**Fig. 1:**
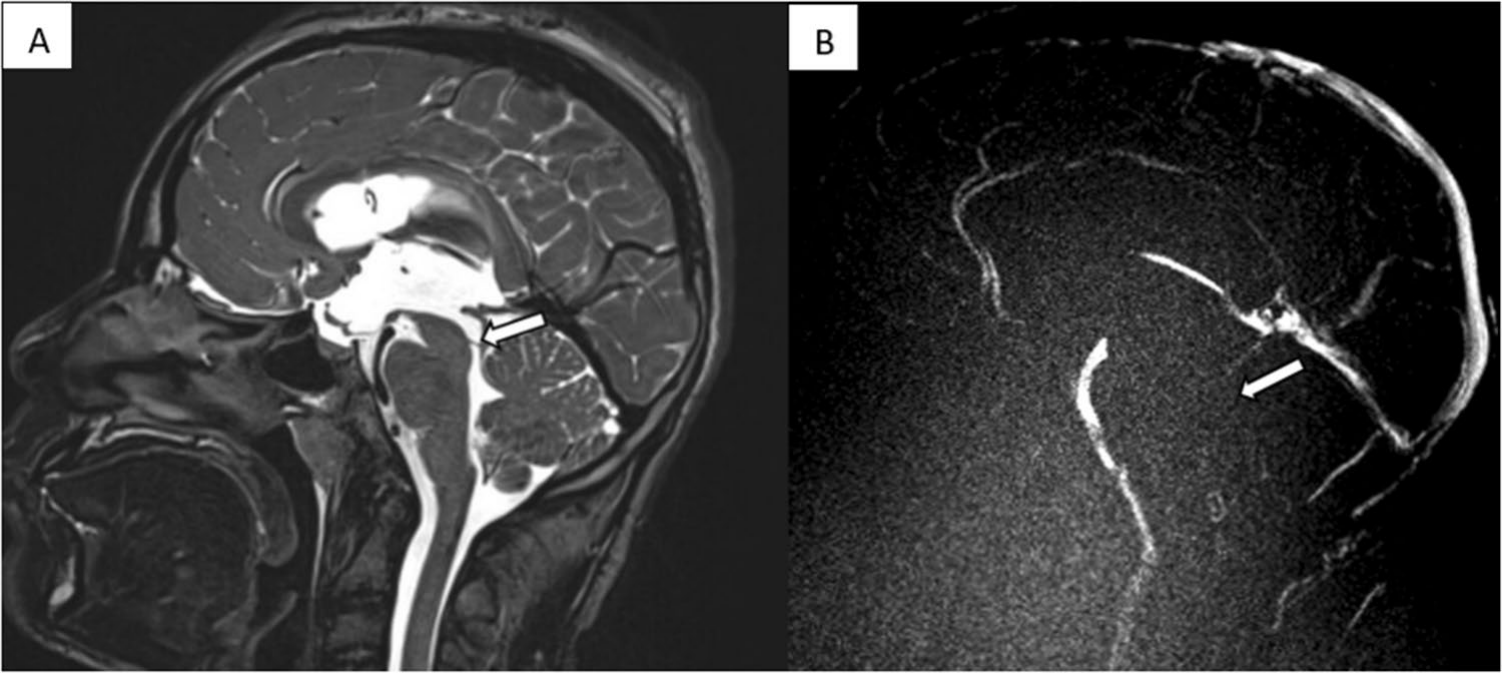
**A** Sagittal T2 TSE image demonstrating aqueductal web. **B** Sagittal phase contrast, MAG sequence, showing the absence of flow across the cerebral aqueduct and 4th ventricle. Flow-related signal at the level of the foramen magnum may be muted due to upstream obstruction

MRI demonstrated an aqueductal obstruction thought to be secondary to a thin transverse web, without contrast enhancement (arrow shown in Fig. 1A).

**Fig. 2:**
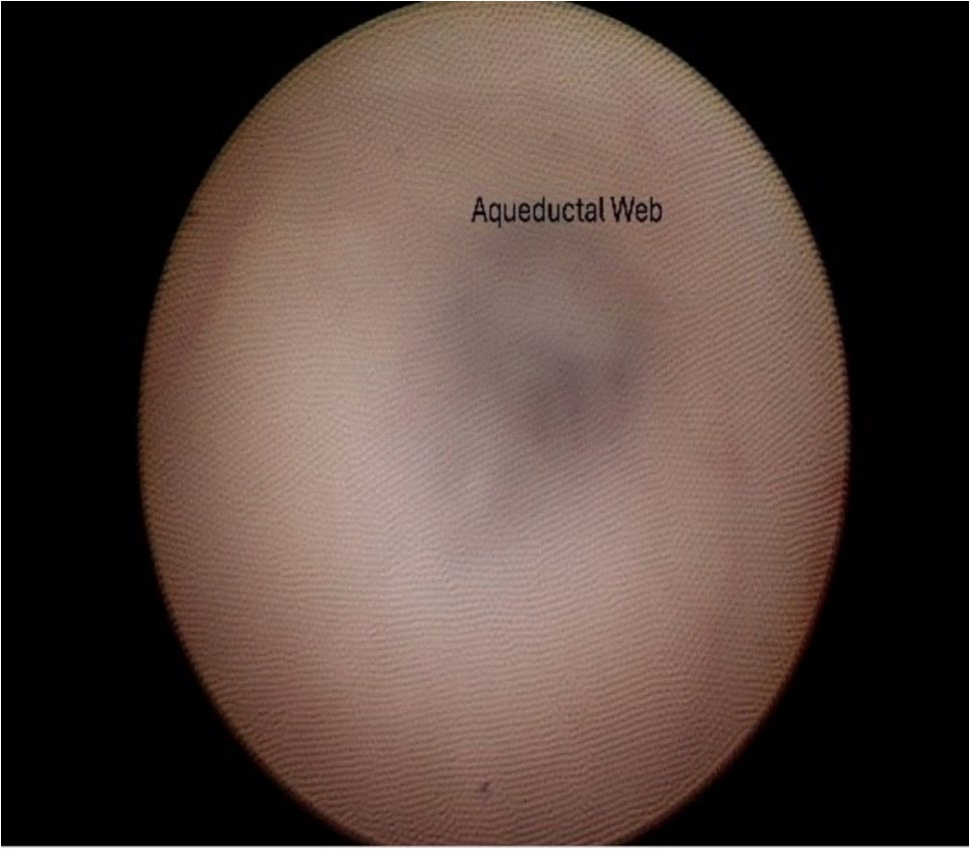
Intra-operative image demonstrating aqueductal web

The patient was taken to the OR for endoscopic exploration and possible aqueductoplasty/fenestration vs. third ventriculostomy. Direct intra-operative visualization within the aqueduct revealed a thickened, gliotic membrane distal to the massa intermedia (shown in Fig. 2 and video). Gentle irrigation and forward pressure were applied to the membrane-like tissue; however, it was deemed unsafe to complete the opening due to the steep angle of the web and the proximity of the dorsal midbrain below and the tectal plate above. As such, the web fenestration was aborted and a standard ETV was performed.

**Fig. 3:**
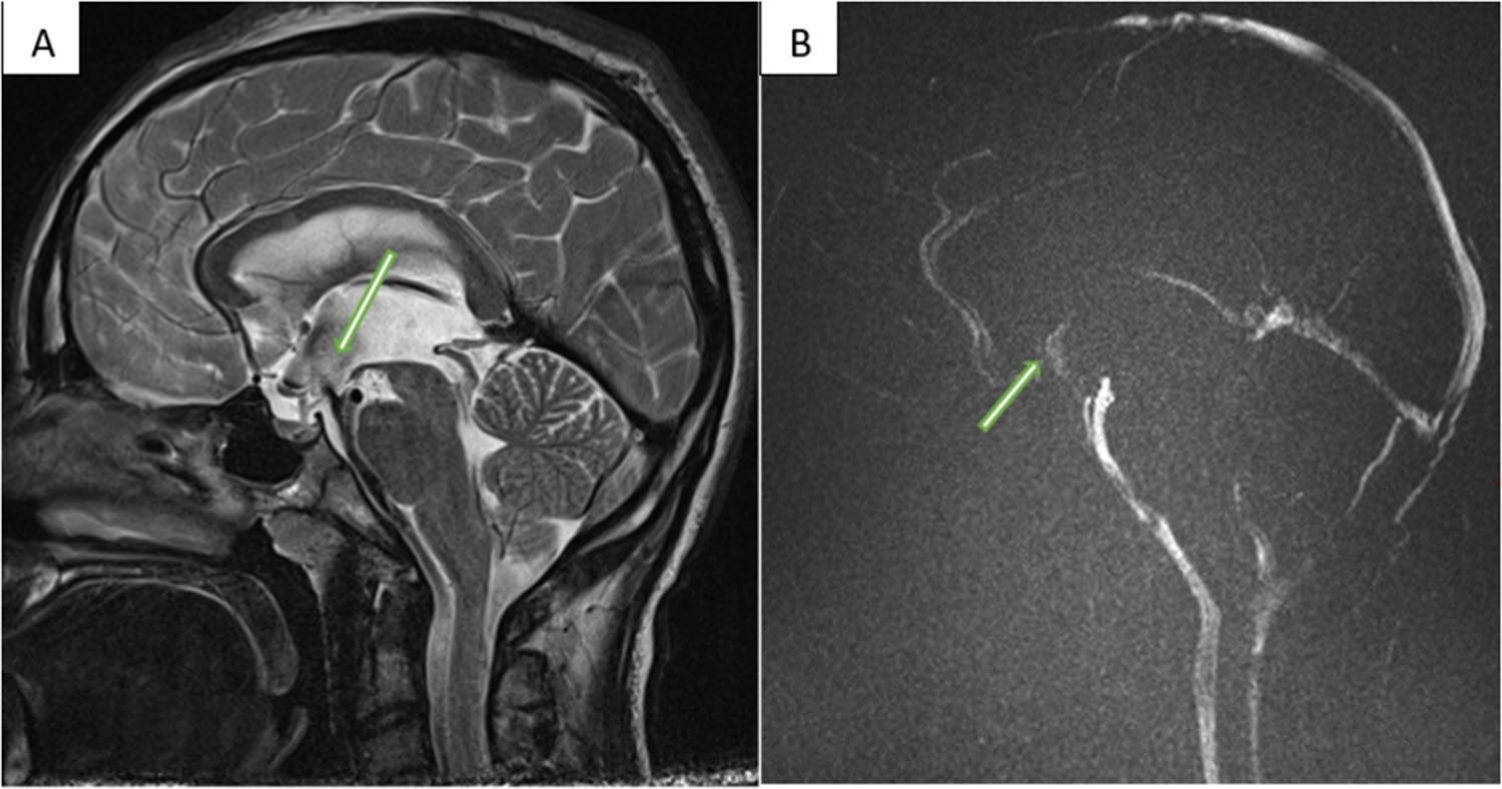
**A** Sag T2 TSE following surgery showing, black flow dephasing artifact as CSF crosses the ETV at the floor of the third ventricle. **B** Sag phase contrast, MAG sequence, showing white flow across the ETV and increased CSF flow about the foramen magnum. Retrograde flow into the 4th ventricle is now visualized

**Fig. 4:**
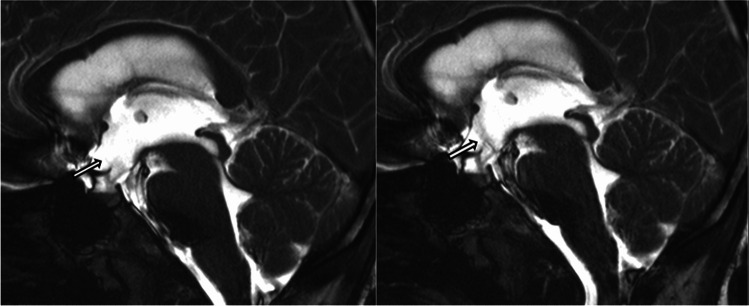
Sag T2 cine TrueFISP images demonstrate stability of the 3rd ventricular floor with pulsatile CSF flow across the patent ETV

Post-operative MRI, on post-operative day 2, revealed a patent ventriculostomy with brisk flow into the prepontine cistern (shown in Figs. 3 and 4). The patient tolerated the procedure well and remained asymptomatic without any new deficits.

## Discussion

Aqueductal stenosis or obstruction is a common cause of obstructive hydrocephalus. The most common etiologies include infection, hemorrhage, idiopathic or genetic congenital stenosis, pineal region tumors, and cerebral vascular malformations [1].

Aqueductal webs are rare lesions leading to obstructive hydrocephalus, their exact prevalence is difficult to determine due to their variable severity and the fact that they are often asymptomatic. While the onset of symptomatic hydrocephalus typically occurs early in life, compensatory mechanisms may enable more prolonged asymptomatic periods lasting into adulthood [2].

Diagnosis via high-resolution imaging is necessary for definitive management. MRI is the modality of choice for assessing aqueductal obstruction, and sagittal T2 sequences are an appropriate sequence to reveal the presence of an aqueductal web [3].

Phase-contrast CSF flow imaging and true fast imaging with steady-state precession (TrueFISP) sequence represent complementary MRI sequences that can be helpful in the evaluation of CSF flow obstructions. TrueFISP allows for cinematic imaging of tissue pulsatility and fluid dephasing. In the setting of an obstructed cerebral aqueduct, relative increases in lateral and third ventricular pressures cause outward-convex bowing of the floor of the third ventricle and the lamina terminalis. Dynamic TrueFISP imaging allows for visualization of the pulsatility of these membranes, which may suggest elevated pressure differentials between the third ventricle and the extra-axial subarachnoid compartment [4]. Phase-contrast imaging has been clinically implemented for many years now and offers direct visualization of CSF flow. This may be used to confirm non-passage of fluid at an obstruction, hyper-dynamic to-and-fro flow upstream to the obstruction, or jet-stream flow across a tight stenosis. When performed orthogonally to the direction of flow, CSF velocity and flow may also be quantified, allowing for a panel of more objective metrics to follow before and after shunting or flow-diversion.

Cerebrospinal fluid (CSF) diversion is the main treatment for obstructive hydrocephalus caused by aqueductal web. Neurosurgical options include endoscopic aqueductoplasty (EAP), endoscopic third ventriculostomy (ETV), and ventriculoperitoneal shunt (VPS).

Endoscopic aqueductoplasty (EAP) can restore the physiological circulation of the CSF in patients with obstructive hydrocephalus caused by aqueductal stenosis. However, given the risk profile, this procedure must be offered after careful patient selection [5]. Furthermore, long-term follow-up is necessary to evaluate patency due to risk of restenosis [6].

ETV is a procedure in which third ventricular (or distal) outflow obstruction is circumvented via an opening in the tuber cinereum such that the prepontine cistern and third ventricle are communicated. ETV success depends on patient age, site of obstruction, and etiology, and it is generally considered the preferred treatment for aqueductal stenosis in children older than 10 years [7]. Based on a meta-analysis of randomized controlled trial (RCTs) comparing ETV to VPS [8], the incidence of complications and mortality was higher with the VPS procedure, and therefore, greater benefits can be achieved using ETV when indicated.

Importantly, spontaneous third ventriculostomy is an infrequent situation where there is communication between the base of the third ventricle and the subarachnoid space; hence, it is important to perform CSF flow studies in cases with suspected aqueductal stenosis to prevent the unnecessary application of ETV [9].

In this report, we describe a rare presentation of aqueductal web at a relatively late stage (adolescence). We attempted a web fenestration, given the dilated appearance of the proximal aqueduct on imaging. This technique was aborted intraoperatively, however, due to the thickened nature of the web and its proximity to the tectal plate and dorsal midbrain. A successful ostomy in the floor of the third ventricle was instead performed, and the patient experienced immediate relief of headaches and vomiting.

## Data Availability

All data generated or analyzed during this study are included in this article. Further enquiries can be directed to the corresponding author.
